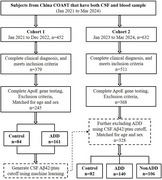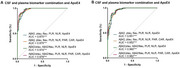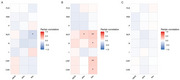# Plasma inflammation biomarkers increase precision for the diagnosis and differential diagnosis of Alzheimer's disease dementia

**DOI:** 10.1002/alz70856_096699

**Published:** 2025-12-24

**Authors:** Meina Quan, Yazhen Huang, Bote Zhao, Shuangshuang Hou, Hong Liu, Qi Wang, Tingting Li, Hongmei Jin, Jianping Jia, Wei Wang

**Affiliations:** ^1^ Innovation Center for Neurological Disorders and Department of Neurology, Xuanwu Hospital, Capital Medical University, Beijing, Beijing, China; ^2^ National Center for Neurological Disorders and National Clinical Research Center for Geriatric Diseases, Beijing, Beijing, China; ^3^ Center of Alzheimer's Disease, Beijing Institute of Brain Disorders, Collaborative Innovation Center for Brain Disorders, Beijing, Beijing, China; ^4^ Beijing Key Laboratory of Geriatric Cognitive Disorders, Clinical Center for Neurodegenerative Disease and Memory Impairment, Beijing, Beijing, China; ^5^ Shandong University of Traditional Chinese Medicine Affiliated Hospital, Jinan, Shandong, China; ^6^ Department of Neurology, the 7th hospital of Dezhou City, Dezhou, Shandong, China

## Abstract

**Background:**

This study aims to explore plasma inflammation biomarkers in Alzheimer's disease dementia (ADD) and dementia of other neurodegenerative subtypes (NonADD), and associations with CSF AD biomarkers.

**Method:**

245 participants including controls and ADD, and 328 participants including controls, ADD and NonADD, were selected from cohort 1 and 2, respectively (Figure 1). CSF biomarkers (Aβ42, ptau181, ttau) and plasma inflammatory biomarkers including counts of neutrophil, lymphocyte, platelet, CRP, Albumin were measured. Statistics were performed to assess prediction performances and correlations.

**Result:**

Combination of two CSF ratios, four inflammation ratios and ApoE4 showed optimal performance in distinguishing ADD from controls (Figure 2A). Combination of two CSF biomarker ratios, two inflammation ratios (neutrophil‐to‐lymphocyte, NLR and platelet‐to‐lymphocyte, PLR) and ApoE4 showed optimal performance in distinguishing ADD from NonADD (Figure 2B). Plasma inflammation biomarkers especially NLR showed positive correlation with CSF AD biomarkers in ADD (Figure 3).

**Conclusion:**

Plasma inflammation biomarkers showed added value in the diagnosis and differential diagnosis of ADD.